# Macrophage in Sporadic Thoracic Aortic Aneurysm and Dissection: Potential Therapeutic and Preventing Target

**DOI:** 10.31083/j.rcm2412340

**Published:** 2023-11-30

**Authors:** Wenyu Song, Guowei Tu, Lieyang Qin, Lai Wei, Jinmiao Chen

**Affiliations:** ^1^Department of Cardiovascular Surgery, Zhongshan Hospital, Fudan University, 200032 Shanghai, China; ^2^Cardiac Intensive Care Center, Zhongshan Hospital, Fudan University, 200032 Shanghai, China

**Keywords:** thoracic aortic aneurysm and dissection, macrophage, inflammation

## Abstract

Thoracic aortic aneurysm and dissection (TAAD) is a life-threatening cardiovascular disorder lacking effective clinical pharmacological therapies. The underlying molecular mechanisms of TAAD still remain elusive with participation of versatile cell types and components including endothelial cells (ECs), smooth muscle cells (SMCs), fibroblasts, immune cells, and the extracellular matrix (ECM). The main pathological features of TAAD include SMC dysfunction, phenotypic switching, and ECM degradation, which is closely associated with inflammation and immune cell infiltration. Among various types of immune cells, macrophages are a distinct participator in the formation and progression of TAAD. In this review, we first highlight the important role of inflammation and immune cell infiltration in TAAD. Furthermore, we discuss the role of macrophages in TAAD from the aspects of macrophage origination, classification, and functions. On the basis of experimental and clinical studies, we summarize key regulators of macrophages in TAAD. Finally, we review how targeting macrophages can reduce TAAD in murine models. A better understanding of the molecular and cellular mechanisms of TAAD may provide novel insights into preventing and treating the condition.

## 1. Introduction

Thoracic aortic aneurysm and dissection (TAAD) is a life-threatening cardiovascular disease with a high mortality rate. Although, the annual incidence of TAAD remains as low as 6 to 16 per 100,000, and it accounts for 1%–2% of all death according to population-based studies [1]. The degradation of elastic fibers and medial degeneration lead to progressive weakening and dilation of the thoracic aorta, which increases the risk of acute aortic dissection or rupture [2]. Surgical repair still remains a guideline-recommended therapy for TAAD [1]. However, open surgical repair for TAAD is a challenge for both medical staff and patients themselves. An effective drug is still lacking to prevent or even reverse TAAD in clinical practice.

The etiology for TAAD is still elusive, with the participation of both genetic and acquired risk factors [3]. According to the latest guidelines, TAAD is classified into three main categories including hereditary, sporadic, and bicuspid aortic valve (BAV)-associated TAAD [1]. Hereditary TAAD accounts for 20% of TAAD and refers to those TAAD patients with clear genetic mutations including Marfan’s syndrome, Loyes-Ditez syndrome, and others [4]. Genetic disorders involve genes encoding various components of the TGF-β signaling cascade (*FBN1*, *TGFBR1*, *TGFBR2*, *TGFB2*, *TGFB3*, *SMAD2*, *SMAD3* and *SKI*) and the smooth muscle contractile apparatus (*ACTA2*, *MYH11*, *MYLK*, and *PRKG1*) [3]. These genetic dysfunctions could directly lead to vasculopathies. In addition, BAV-associated TAAD is a compounding pathological process with both participation of hereditary (i.e., *NOTCH1*, *ACTA2*, *MAT2A*, *SMAD6*, and *LOX*) and acquired hemodynamics factors [5]. Hereditary and BAV-associated TAAD have previously been reviewed and thus not considered in this review [4, 6]. The TAAD referred to in this article focuses on sporadic TAAD without a hereditary basis or BAV.

The molecular mechanisms of TAAD are complicated biological processes with the involvement of various cell types including immune cells [7]. Macrophages are one of the major immune cells increased in TAAD both in human and murine models. Aggregation of macrophages is critical for thoracic aortic weakening, dilation, aneurysm, and dissection. This review will first introduce the important role of inflammation and immune cell infiltration in the pathogenesis of TAAD. Then, we will systematically summarize the role of macrophages in thoracic aortic dilation, aneurysm, and dissection. We further focus on how to regulate macrophages in TAAD. Finally, we will discuss the translational prospect of targeting macrophages pharmacologically to reduce TAAD.

## 2. Structure of Normal Thoracic Aortic Walls and Basic Pathophysiology of TAAD

**Fig. 1.**
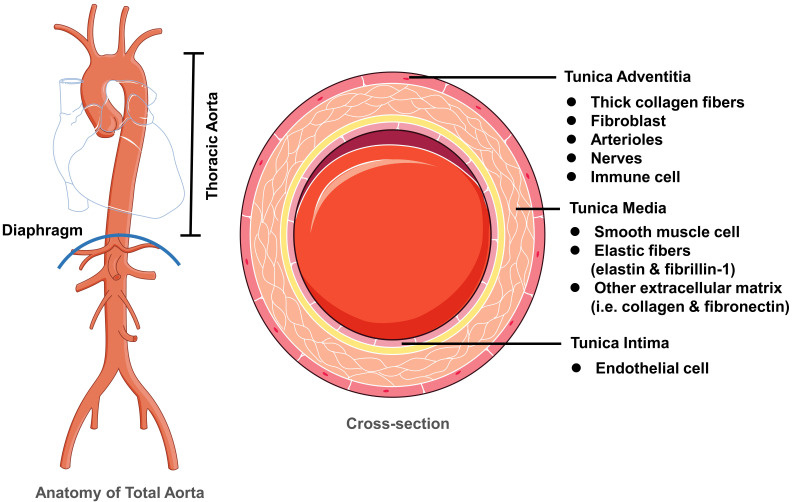
**Normal structure of thoracic aortic wall**. Normal thoracic aortic walls are composed of three layers including tunica intima, tunica media and tunica adventitia. The tunica intima is mainly composed of endothelial cells. The tunica media mostly contains smooth muscle cells, elastic fibers, and other ECM components. The tunica adventitia is mainly composed of thick collagen fibers, fibroblast, arterioles, nerves, and various types of immune cells. Crosslinks of elastic fiber-ECM, SMC-ECM and SMC-SMC maintains normal strength of thoracic aortic walls and prevent thoracic aorta from dilation, aneurysm, and dissection. ECM, extracellular matrix; SMC, smooth muscle cell.

Normal thoracic aortic walls are composed of tunica intima, media and adventitia (Fig. 1) [8]. Tunica intima is mainly composed of endothelial cells (ECs). Tunica media forms the main structure of thoracic aortic walls, comprising smooth muscle cells (SMCs), elastic fibers (fibrillin-1 and elastin) and many other extracellular matrix (ECM) proteins. The tunica adventitia is mainly composed of thick collagen fibers with fibroblasts to maintain its integrity, which form the external cuff. The adventitia also contains arterioles, nerves, and is a major source of aortic immune cells. Of note, the vasa vasorum is mainly found in the adventitia, but also exists in the outer layers of the media to feed the aorta. This vascular bed is actually the main source of the inflammatory cells in the outer wall media/adventitia [9]. Crosslinks of elastic fiber-ECM, SMC-ECM and SMC-SMC maintains normal strength of thoracic aortic walls, suppress immune cell infiltration, and prevent the thoracic aorta from weakening.

**Fig. 2.**
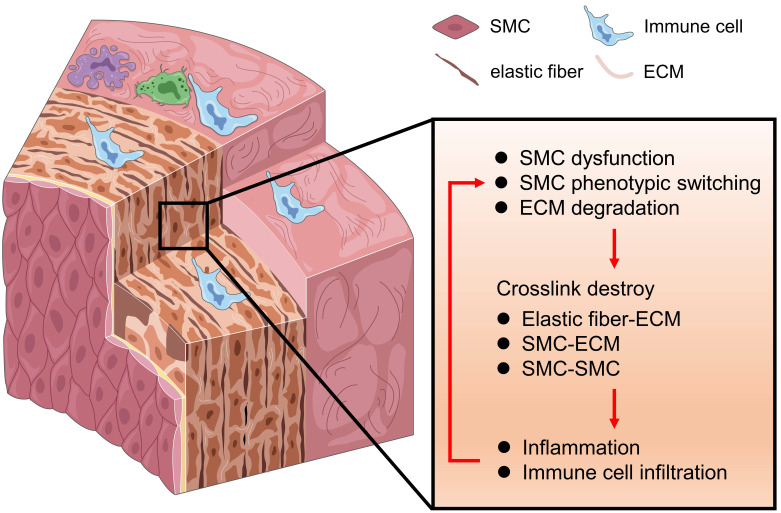
**The basic pathophysiology of thoracic aortic aneurysm and dissection (TAAD)**. Medial degeneration and degradation of elastic fibers are basic hallmarks of TAAD. Elastic fiber-ECM, SMC-ECM, and SMC-SMC crosstalk are destroyed due to SMC dysfunction, phenotypic switching, and ECM degradation. When immune cells infiltrate into the media, it may lead to medial inflammation, SMC phenotypic switching, SMC dysfunction and ECM disruption. These biological processes form a positive feedback loop. SMC, smooth muscle cell; ECM, extracellular matrix.

The pathogenesis of TAAD is a progressive biological process with the involvement of various aortic cell types and components including SMCs, ECs, myofibroblasts, immune cells and the ECM. Medial degeneration and degradation of elastic fibers are basic morphological and pathological characteristics of TAAD. Elastic fiber-ECM, SMC-ECM, and SMC-SMC crosstalk are destroyed due to SMC dysfunction, phenotypic switching, and ECM degradation. During these pathological processes, immune cells play a pivotal role. When immune cells from the adventitia infiltrate into the media-intima, it may lead to medial inflammation, degeneration, SMC phenotypic switching, dysfunction, and ECM disruption. In turn, inflammatory cells could recruit secondary to ECM disruption and initiate a positive feedback loop (Fig. 2). These biological processes further contribute to weakness and vulnerability of thoracic aortic walls, leading to progressive thoracic aortic dilation, aneurysm, and dissection.

## 3. Inflammation and Immune Cell Infiltration is a Critical Hallmark of TAAD

There is adequate evidence showing that inflammation and immune cell infiltration are important hallmarks of TAAD based on clinical specimens [7, 10, 11, 12, 13, 14, 15, 16, 17, 18, 19]. Inflammation associated proteins are dramatically increased in TAAD marked by *IL-1β*, *IL-11*, *IL-22*, *INF-γ*, *IgG4*, *CX3CR1* and *HBB * [20, 21, 22, 23], accompanied with immune cell infiltration [24]. Single-cell transcriptomic analysis and histological evidence proved that macrophages, natural killer cells (NK cells), T cells, mast cells and neutrophils increased in the intima-media of TAAD [24, 25, 26, 27].

Parallel with findings in humans, thoracic aortic specimens of TAAD mice models are also characterized by inflammation and immune cell infiltration. Currently, TAAD mice models are established mostly by β-aminopropionitrile (BAPN) administration, direct elastase treatment to thoracic aorta, angiotensin Ⅱ (Ang II) or combined administration of BAPN and infusion of Ang II [28]. The inflammatory response is significantly activated in thoracic aortic specimens of BAPN induced TAAD mice, marked by an increase of *IL-1β*, *IL-3*, *IL-5* and *IL-18 * [29, 30, 31, 32], with significant recruitment of immune cell in the adventitia [33, 34]. In addition, inflammatory response and immune cell recruitment were also observed in Ang II induced TAAD [35, 36, 37].

## 4. Macrophage in Thoracic Aortic Dilation, Aneurysm and Dissection

### 4.1 Origin of Macrophage in the Aortic Wall

Although different types of immune cells were increased in thoracic specimens of TAAD, macrophages are one of the most abundant types [27, 34, 38, 39]. Macrophages found in the thoracic aortic wall are mainly derived from two main sources. Most macrophages in the aneurysmal thoracic aortic wall derive from circulating monocytes, which are produced from bone barrow and mobilized from peripheral reservoirs such as the spleen [40, 41, 42]. Transplantation of bone marrow cells expressing green fluorescent protein revealed that a proportion of macrophages in the aortic adventitia originated from bone marrow-derived monocytes [43]. Besides circulating monocytes, aortic macrophages might also develop from embryonic precursors and early postnatal circulating monocytes. This group of macrophages are known as tissue-resident macrophages, which are independent from bone-marrow progenitors and are self-maintained and self-developed during adulthood [41, 42]. Updated investigations have added evidence that tissue-resident macrophages might also originate from multipotent stem cells, which was identified in the media and adventitia of TAAD thoracic aorta expressing macrophage marker *CD68* [44, 45].

### 4.2 Classification of Macrophage in TAAD

Macrophages have been classified into several different phenotypes over the past two decades, mainly including M1 and M2 macrophages [46]. M1 macrophages are also known as pro-inflammatory macrophages with production of proteolytic enzymes and pro-inflammatory cytokines. In comparison, M2 macrophages have an anti-inflammatory role through promoting ECM remodeling and tissue repair.

**Fig. 3.**
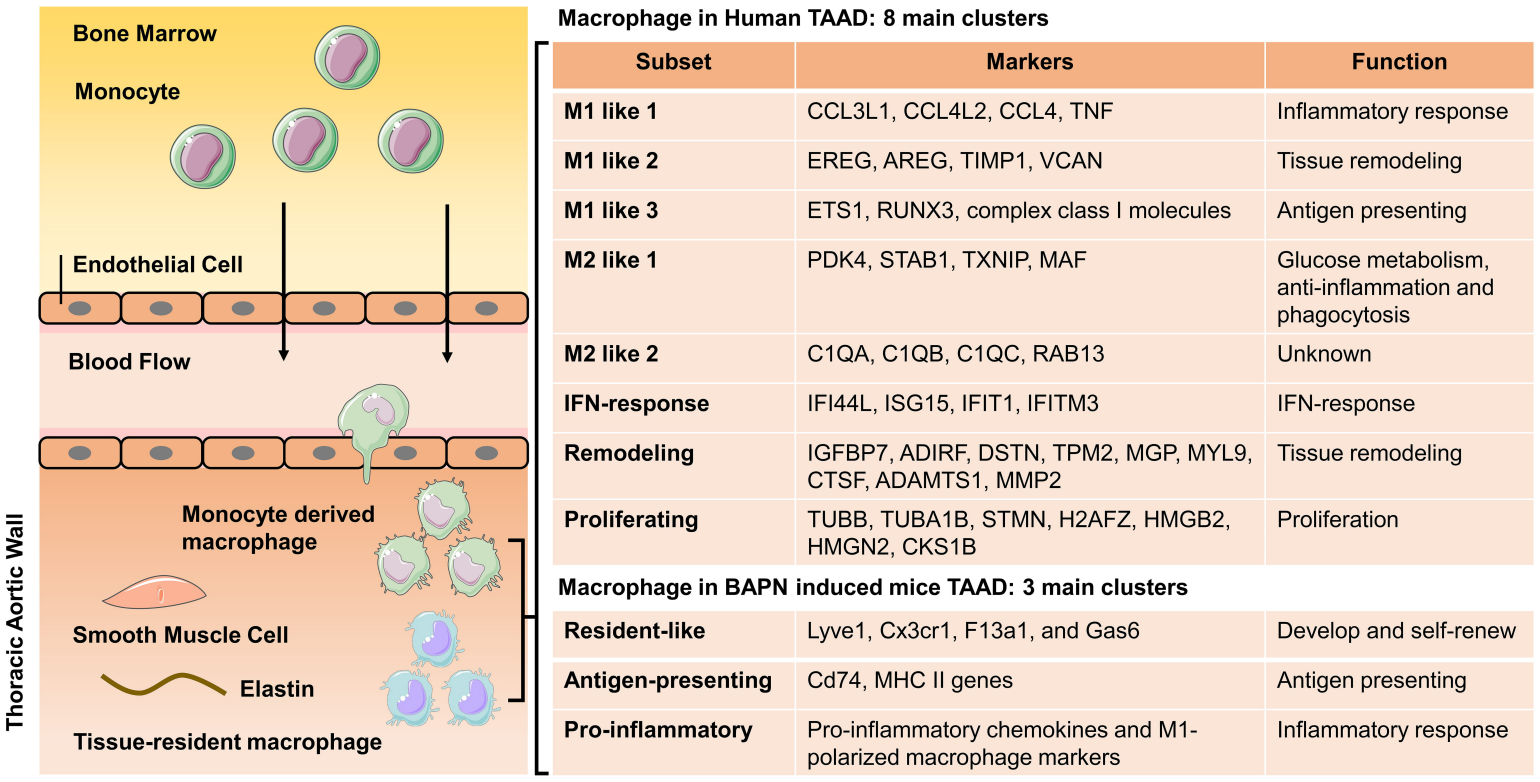
**Origin and classification of macrophages in TAAD thoracic aortic wall**. Macrophages within thoracic aortic walls originate mainly from two sources. One main origin of macrophages is circulating monocytes. Another source is embryonic precursors and early postnatal circulating monocytes. Macrophages from human aneurysmal thoracic aortas are classified into eight subclusters including M1 like 1, M1 like 2, M1 like 3, M2 like 1, M2 like 2, *IFN*-response, remodeling, and proliferating macrophage. Macrophages of mice aneurysmal thoracic aorta induced by BAPN are divided into three subgroups including resident-like, antigen-presenting and pro-inflammatory macrophage. TAAD, thoracic aortic aneurysm and dissection; *IFN*, interferon; *TNF*, tumor necrosis factor; BAPN, β-aminopropionitrile; *MHC*, major histocompatibility complex; TAAD, thoracic aortic aneurysm and dissection.

Single-cell RNA sequencing described a systemic landscape of immune cells in TAAD. Macrophages were classified into ‘M1 like’, ‘M2 like’, ‘IFN-response’, ‘remodeling’, and ‘proliferating’ subgroups [27, 47, 48] (Fig. 3). ‘M1 like’ macrophages were identified by *IL-1B*, *TNF* and *NFKB1*, while ‘M2 like’ macrophages were characterized by *MERTK*, *MRC1*, *STAB1* and *CD163*. ‘M1 like’ macrophages are further divided into three subgroups including ‘M1 like 1’, ‘M1 like 2’ and ‘M1 like 3’. ‘M1 like 1’ macrophages expressed several cytokine genes, such as *CCL3L1*, *CCL4L2*, *CCL4* and *TNF*, associated with the inflammatory response. ‘M1 like 2’ macrophages expressed *EREG*, *AREG*, *TIMP1* and *VCAN* which are involved in tissue remodeling and the inflammatory response. ‘M1 like 3’ macrophages expressed *ETS1*, *RUNX3* and genes encoding major histocompatibility complex (MHC) class I molecules. This result indicated that ‘M1 like 3’ macrophages could present antigens to ${\mathrm{CD8}}^{+}$ T lymphocytes. Similarly, ‘M2 like’ macrophages were classified into two subgroups including ‘M2 like 1’ and ‘M2 like 2’. ‘M2 like 1’ macrophages expressed *PDK4*, *STAB1*, *TXNIP* and *MAF*, involved in glucose metabolism, anti-inflammation, and phagocytosis. ‘M2 like 2’ macrophages expressed *C1QA*, *C1QB*, *C1QC* and *RAB13*. ‘IFN-response’ macrophages expressed interferon-induced genes, including *IFI44L*, *ISG15*, *IFIT1* and *IFITM3*. ‘Remodeling’ macrophages shared characteristics with SMCs and fibroblasts including *IGFBP7*, *ADIRF*, *DSTN*, *TPM2*, *MGP*, and *MYL9*. In addition, protease genes including *ADAMTS1*, *MMP-2* and *CTSF* were highly expressed, associated with tissue remodeling. Finally, ‘proliferating’ macrophages are closely associated with proliferation with high expression of microtubule-related genes *TUBB*, *TUBA1B* and *STMN*, histone-related genes *H2AFZ*, *HMGB2* and *HMGN2* and cyclin-dependent kinases regulatory subunit 1 *CKS1B*.

Macrophage subgroups are also identified in the thoracic aorta of BAPN administrated TAAD mice. A total of three macrophage subgroups were identified including ‘resident-like’, ‘antigen-presenting’ and ‘pro-inflammatory’ subsets [34]. In detail, ‘resident-like’ macrophages highly expressed *Lyve1*, *Cx3cr1*, *F13a1*, *Lyve1*, and *Gas6*. This group of macrophages were thought to be located residentially in the adventitia and may develop and self-renew independently of *CCR2*-mediated monocyte recruitment [49, 50]. ‘Antigen-presenting’ macrophages highly expressed *Cd74*, *MHC II *genes. This may indicate that this macrophage subcluster is involved in presenting antigens. ‘Pro-inflammatory’ macrophages highly expressed pro-inflammatory chemokines and M1-polarized macrophage markers. This subcluster is similar to M1 macrophages and may infiltrate and drive inflammatory responses. Subclusters, classifications and definitions of these new macrophage subgroups might provide novel insights into understanding the pathogenesis of TAAD.

### 4.3 The Role of Macrophage in TAAD

#### 4.3.1 Infiltration into the Media Layer of Thoracic Aortic Wall

**Fig. 4.**
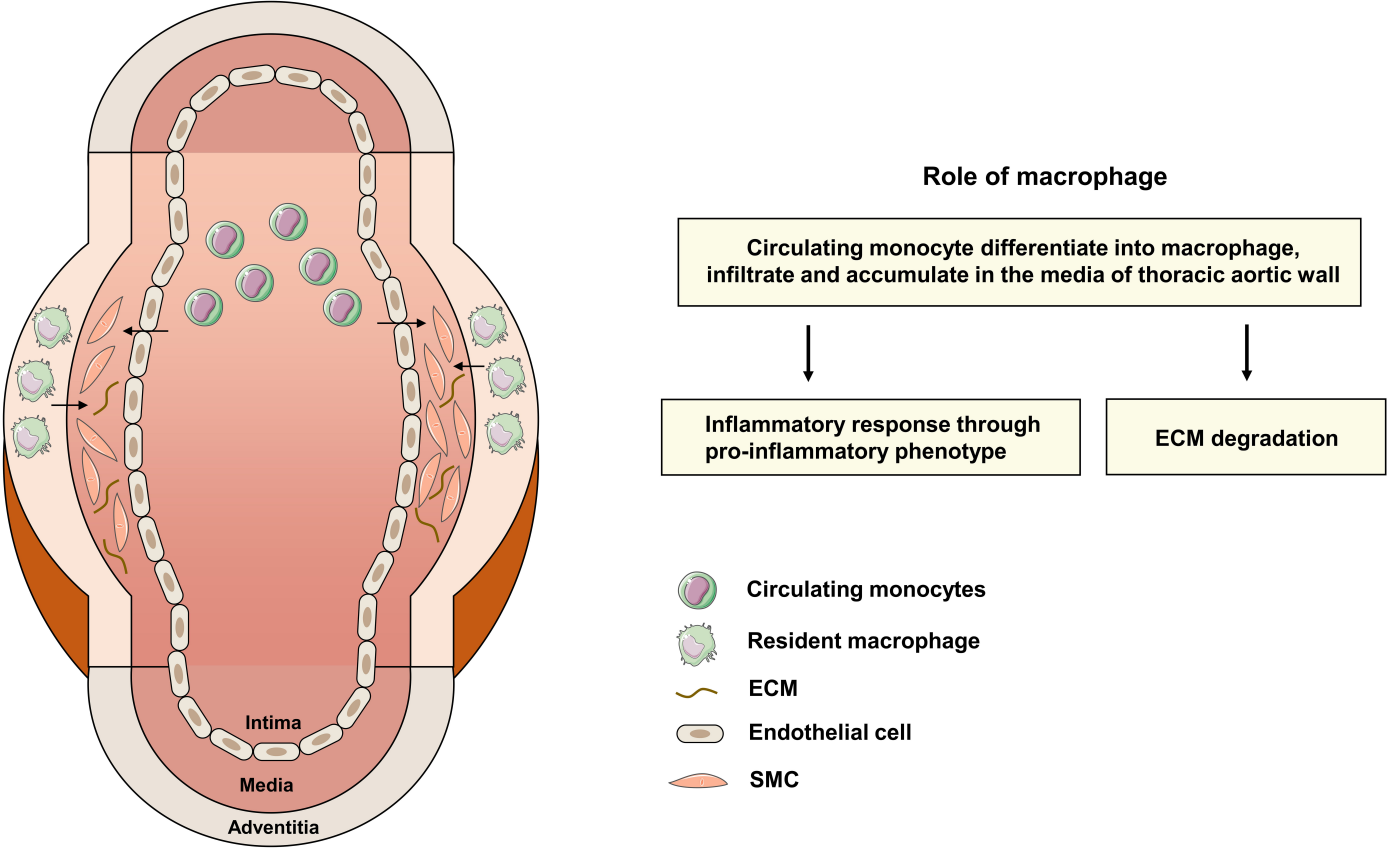
**The Role of Macrophages in the Pathogenesis of TAAD.** Macrophages play a pivotal role in the pathogenesis of TAAD both through the inflammatory response and ECM degradation. Circulating monocytes first differentiate into macrophages, infiltrate, and accumulate in the media layer of the thoracic aorta wall. They further exert an inflammatory response through pro-inflammatory phenotypes and produce *MMPs* and *ADAMTSs* for ECM degradation. TAAD, thoracic aortic aneurysm and dissection; ECM, extracellular matrix; SMC, smooth muscle cell; *MMP*, matrix metalloproteinase; *ADAMTS*, ADAM metallopeptidase with thrombospondin type 1 motif.

In the pathological process of TAAD, macrophages infiltrate from the adventitia into the media and participate in the inflammatory response, ECM degradation and medial degeneration [51] (Fig. 4). With the assistance of splenic B lymphocytes, monocytes mobilize from the spleen and infiltrate into the aortic walls [52, 53]. Subsequently, *CCL2* and *IL-6* produced by adventitia fibroblasts initiate monocyte recruitment and differentiation into macrophages, followed by stimulation of fibroblast proliferation and more production of *CCL2* and *IL-6* to form a positive feedback loop [54]. In the inflammatory microenvironment of the aortic media, macrophages further accumulate and activate with the assistance of inflammatory cytokines, chemokines, and other biochemical factors such as reactive oxygen species [55]. Although tissue-resident macrophages only occupy a small proportion, their role should not be overlooked and requires future investigation.

#### 4.3.2 Inflammatory Response

Macrophage infiltration and accumulation initiates an amplified inflammatory response and ECM degradation, leading to disintegration and destruction of elastic fiber-ECM, SMC-ECM, and SMC-SMC crosslinks. Macrophages are prominent inflammatory signal senders on SMC in TAAD through *CXCL*, *CCL*, *TNF*, *IFN-II*, *IL-16* and complement pathways [56, 57]. Consolidated evidence has shown that SMC is a major target cell of macrophages in TAAD. Cell-interaction analysis has also highlighted increased communication between macrophages and T cells in TAAD tissues, indicating that macrophages might drive further inflammatory responses on SMCs through other intermediary cells [58].

Pro-inflammatory macrophages could both produce and respond to inflammatory mediators, therefore further invade into thoracic aortic walls, and trigger inflammatory response through positive feedback loops [59, 60, 61]. One possible theory proposes that aneurysmal and dissected thoracic aortic walls are the main sources of *CXCL1*, which could further induce neutrophils to produce *IL-6 * [62]. Increased *IL-6* might then induce pro-inflammatory macrophages to secrete *CCL2*, which further promotes macrophage activation and invasion [63].

#### 4.3.3 ECM Degradation

ECM in the aortic media plays a distinct role in preventing TAAD as well. When ECM degrades, thoracic aortic walls may lose their integrity, and begin to weaken and dilate. Matrix metalloproteinases (*MMPs*) and ADAM metallopeptidase with thrombospondin type 1 motifs (*ADAMTS*) are known to have the capability to degrade various components of ECM for aortic vulnerability [64]. Importantly, *MMPs* and *ADAMTSs* were mainly expressed in macrophages [32, 38, 65, 66, 67]. This evidence supports the idea that macrophage-derived *MMPs* and *ADAMTSs* may exert an important effect on ECM degradation.

## 5. Identification of Key Regulators of Macrophages in TAAD

### 5.1 Regulators of Macrophage Accumulation and Infiltration

Since macrophages play such a pivotal role in the pathogenesis of TAAD, understanding the regulatory mechanism for macrophages might provide novel insights into macrophage-based therapy for TAAD [68]. Over the decades, a series of regulators of macrophages have been identified as being closely associated with the pathogenesis of TAAD. These regulators are mainly classified into three categories. The first type of regulators affects macrophage accumulation and infiltration, which initiate further biological process. The remaining two types of regulators influence macrophage functions through modulating pro-inflammatory phenotype and *MMPs* expression (Table 1, Ref. [11, 29, 32, 34, 35, 63, 65, 69, 70, 71, 72, 73, 74, 75, 76, 77, 78, 79, 80, 81, 82, 83, 84, 85, 86, 87, 88, 89, 90, 91, 92, 93]).

**Table 1.** **Potential Regulators of Macrophages in TAAD**.

Mechanisms	Regulators
Macrophage Accumulation and Infiltration	*CCL2*/*CCR2 * [63, 69, 70], *IL-1β*/*IL-1R * [29], *IL-6 * [35], *SMAD3 * [71], *ADAMTS-4 * [72], *CCN4 * [73], microRNAs (*miR-146a*, *miR-21*, *miR-29b*, *miR-29c* and *miR-27b*) [74]
Pro-inflammatory Phenotype and Inflammatory Response	*LRP1 * [75], *BMAL1 * [76], *TGF-β* [77, 79], *ANGPTL8 * [11], *AT1R * [78], *NEU1 * [80], *ADAMTS1 * [81], *SR-A1 * [82], *SOCS3 * [83, 84], *RGS1 * [85], *IL1RN * [34], *TREM1 * [34], C18-ceramide [86], succinate [87], tryptophan [88], kynurenine [88], quinolinic acid [88]
Macrophage-based ECM Degradation	*LGMN * [89], urokinase-generated plasmin [90], C18-ceramide [86], *HIF-1α* [91], *MCP-1 * [63], *IL-6 * [63], cytosolic DNA [65], *STING * [65], *NLRP3 * [92], *IL-3*/*IL-3Rβ* [32], *miR-320 * [93]

TAAD, thoracic aortic aneurysm and dissection; ECM, extracellular matrix.

Previous investigations have uncovered some mechanisms that are involved in macrophage accumulation, infiltration, driving the inflammatory response. Macrophage infiltration was more significant in TAAD patients with atherosclerosis [94]. Elevated plasma levels of LDL cholesterol promoted Ang II-induced TAAD through enhancing macrophage infiltration [95]. Nicotine free base promoted TAAD progression in SMC specific *TGFBR2* knockout mice, whose effect was sensitized by BAPN co-stimulation [96]. These results might partly explain why atherosclerosis, hypercholesterolemia and tobacco use might serve as established risk factors for TAAD [97]. However, these studies only provided observations on this phenomenon and did not provide in-depth mechanisms of how macrophages differentiate and infiltrate into thoracic aortic walls.

As mentioned above, circulating monocytes are the main sources of thoracic aortic macrophages. They might recruit and infiltrate into aortic walls through chemokine/chemokine-receptor pathways and selectins [63, 98, 99, 100, 101, 102]. The *CCL2*/*CCR2* axis is confirmed as a critical manner of the chemokine/chemokine-receptor pathway that participate in macrophage accumulation and infiltration into aortic walls. Global and myeloid specific deficiency of *CCR2* decreased macrophage recruitment, inhibited inflammatory cytokines, and reduced Ang II-induced aortic dissection in mice [63]. Similarly, global and bone-marrow-derived cell-specific *CCL2* deficiency protected mice from elastase-induced aneurysms [69, 70]. Besides *CCL2* and *CCR2*, genetic knockout of *IL-1β*, *IL-1R*, *IL-6*, *SMAD3*, *ADAMTS-4* or *CCN4* attenuated thoracic aortic dilation through inhibition of macrophage recruitment [29, 35, 71, 72, 73]. Updated knowledge has identified that epigenetic mechanisms may also participate in the regulation of macrophage infiltration [103]. Upregulation of *miR-146a* and *miR-21*, as well as the downregulation of *miR-29b*, *miR-29c* and *miR-27b* are closely associated with aortic inflammation and macrophage infiltration [74]. These miRNAs might be under the control of mesenchymal stem cells as immunomodulators of aortic inflammation through regulation of proinflammatory cytokines [45, 104, 105, 106].

Exogenous administration of specific drugs could also reduce the pathologic changes in different mouse models of TAAD through reducing macrophage infiltration and inflammation. Pretreatment of *IL-1R* antagonist anakinra reduced macrophage infiltration and attenuated TAAD formation induced by elastase [29]. Similarly, calcium channel blocker azelnidipine reduced BAPN-induced TAAD through anti-inflammatory effects [107]. Several investigations focus on in-depth mechanisms of how macrophage infiltration is reduced. The glycolytic enzyme pyruvate kinase M2 activator TEPP-46 markedly attenuated the progression of TAAD induced by BAPN through inhibition of macrophage infiltration associated with the *NOD*-like receptor family and *NLRP3* inflammasome [55].

### 5.2 Regulators of Pro-Inflammatory Phenotype and Inflammatory Response

When macrophages accumulated and infiltrate, the pro-inflammatory phenotype and associated inflammatory response are essential to participate in the pathological process of TAAD. Hypertension is the most important risk factor for TAAD [108, 109]. A previous study has shown that hypertensive TAAD patients tended to have more pro-inflammatory macrophages in the adventitia and media of thoracic aorta [110]. This result may indicate that hypertension might promote a pro-inflammatory phenotype of macrophages, with underlying mechanisms needing further investigation.

A pro-inflammatory phenotype of macrophages in TAAD is mainly regulated through affecting production of inflammatory factors and the capability of migration and invasion of macrophages [111]. Several pro-inflammatory regulators have been identified over the past two decades. Single-nuclear RNA sequencing and genome-wide association studies have identified *LRP1* as a key potential regulator of inflammation in macrophage of TAAD [75]. In experimental investigations, overexpression of core circadian clock gene *BMAL1* induced a pro-inflammatory response in cultured macrophages [76]. *TGF-β* induced severe inflammatory response through enhancing macrophage invasion [77]. Silencing of *ANGPTL8* and *AT1R* decreased inflammatory factors in macrophages including *IL-1β*, *IL-6*, *MCP-1* and *TNF-α* [11, 78]. *In vivo*, myeloid-specific knockout of *TGF-β* or *NEU1* reduced macrophage pro-inflammatory functions and ameliorated TAAD induced by BAPN [79, 80]. *ADAMTS1*-deficient macrophages exhibited low activity of the inflammatory response through abrogated migration capacity in BAPN mice [81].

Meanwhile, some molecules demonstrated anti-inflammatory effects on macrophages in TAAD. Functional silencing or knockout of *SR-A1* or *SOCS3* may aggregate TAAD in murine models. In detail, *SR-A1* deficiency aggravated BAPN induced TAAD in mice through *TYRO3* mediated efferocytosis and inflammatory cascades in macrophages [82]. Macrophage-specific deletion of *SOCS3* exaggerated TAAD through M1-dominant differentiation of macrophages via acute enhancement of *STAT3* activation [83, 84]. Conversely, restoration of *RGS1* reduces Ang II-induced TAAD through inhibiting macrophage chemotaxis and desensitizes chemokine receptor signaling [85].

Notably, metabolic reprogramming is required for the proper polarization and function of activated macrophages [112]. Similar to the Warburg effect of tumor cells, pro-inflammatory M1 pro-inflammatory macrophages increase glucose consumption, lactate release and decreased oxygen consumption rate. However, M2 anti-inflammatory macrophages are characterized by the employment of oxidative glucose metabolism pathways [113]. Besides, fatty acid, vitamin and iron metabolisms are also closely associated with macrophage polarization [114]. Using untargeted metabolomics of clinical TAAD specimens and BAPN-induced mice model, C18-ceramide was identified to be increased through the *de novo* synthesis pathway and promoted macrophage pro-inflammatory phenotype through the *NLRP3*-caspase 1 pathway [86]. Similarly, succinate, tryptophan, kynurenine, quinolinic acid and kynurenine-to-tryptophan ratio were also identified to be increased in TAAD [87, 88]. Knockdown of the key synthetic enzyme of succinate *OGDH* or kynurenine pathway enzyme kynureninase could reduce the expression of inflammatory factors in macrophages.

Regulation of the pro-inflammatory phenotype in macrophages through pharmacological intervention has been proven effective in attenuating TAAD *in vivo*. Targeting pro-inflammatory *${\mathrm{Il1rn}}^{+}$*/*${\mathrm{Trem1}}^{+}$* macrophage subpopulations through *mLR12* could significantly reduce thoracic aortic rupture rate in BAPN-administrated mice [34]. Dexamethasone treatment suppressed *NF-κB* signaling pathway in macrophages and further reduced the inflammatory response, immune cell infiltration and incidence of TAAD in BAPN mice [115]. Selective mineralocorticoid receptor antagonist eplerenone protected mice from BAPN-induced TAAD through decreasing *TNFα* and *IL-6* in macrophages [116]. An in-depth understanding of pro-inflammatory macrophages might broaden our horizon on anti-inflammatory therapy for TAAD.

### 5.3 Regulators of Macrophage-Based ECM Degradation

ECM degradation is another critical mechanism involved in TAAD development, which has been reported to be under the control of specific molecules. For example, macrophage-derived legumain is essential for ECM degradation. Macrophage-specific deletion of legumain (*LGMN*) alleviated BAPN-induced thoracic aortic dilation, aneurysm, and dissection in mice [89]. Deficiency of urokinase-generated plasmin protected against media destruction and aneurysm formation by reducing the activation of pro-*MMPs * [90]. Inhibition of endogenous ceramide synthesis in macrophages by myriocin, attenuated BAPN-induced TAAD in mice through reducing macrophage inflammation and expression of *MMPs * [86].

Specific MMPs in macrophages could be directly regulated, including *MMP-2*, *MMP-9*, and *MMP-12*. macrophage-derived HIF-1α activation triggered ECM degradation and elastic plate breakage through increasing *ADAM17* to induce *MMP-2* and *MMP-9* expression [91]. *In vitro*, *MCP-1* and *IL-6* enriched conditioned medium induced differentiation of monocytes into macrophages and expression of *MMP-9 * [63]. Cytosolic DNA from damaged aortic SMCs induced *MMP-9* expression in macrophages through the *STING* pathway and its target *IRF3 * [65]. STING deficiency protected against thoracic aortic aneurysm and dissection through reducing *MMP-9* in macrophages. Selective *NLRP3* inhibitor MCC950 prevented TAAD through reducing *MMP-9* expression and activation in macrophage via the *NLRP3*-caspase-1 inflammasome [92]. Another study confirmed an *IL-3*/*IL-3Rβ*/*MMP-12* axis in macrophage during the progression of TAAD. *IL-3* deficiency in macrophages diminished *JNK* and *ERK1/2* dependent AP-1 pathways, thus decreasing expression of *MMP-12 * [32]. Epigenetic mechanisms also participate in the regulation of *MMPs* in macrophages, such as *miR-320 * [93].

## 6. Future Prospective: Targeting Macrophage to Reduce TAAD

Currently, no pharmacological therapy has proven effective in preventing and treating TAAD patients in clinical practice. Since macrophages play an essential role in TAAD and could be regulated through versatile mechanisms, it is worthwhile to investigate whether targeting macrophages could reduce TAAD. As mentioned above, several anti-inflammatory drugs may have protective effects against murine TAAD by reducing macrophage infiltration and function (Table 2, Ref. [29, 34, 55, 86, 92, 107, 115, 116]). However, some opposite views state that not all anti-inflammatory drugs have therapeutic and preventive effects against TAAD and may even exert detrimental effects [117, 118]. This phenomenon deserves further investigation because anti-inflammatory therapy might be impacted by different TAAD models or varying signaling cascades. Translation of these protective methods against TAAD might still have a long way to go before being implemented in clinical practice. Future investigations are needed to focus on how to target macrophages to both prevent and treat TAAD both in murine and human TAAD [119].

**Table 2.** **Summary of effective exogenous drugs against murine TAAD via macrophage associated mechanisms**.

Drug	Target	Murine TAAD model	Detailed downstream	Reference
Anakinra	*IL-1R* antagonist	Elastase	Reduce macrophage infiltration and inflammation	Johnston WF [29]
Azelnidipine	Calcium channel blocker	BAPN + Ang II	Reduce macrophage infiltration and inflammation	Kurobe H [107]
TEPP-46	Pyruvate kinase M2 activator	BAPN	Reduce *NLRP3* inflammasome-mediated *IL-1β*secretion and macrophage infiltration	Le S [55]
mLR12	*Trem1* blocker	BAPN	Reduce macrophage infiltration and inflammation	Liu X [34]
Dexamethasone	Synthetic glucocorticoid	BAPN	Suppress *NF-κB* signaling in macrophage	Wang X [115]
Eplerenone	Selective *MR* antagonist	BAPN + Ang II	Suppress *TNFα* and *IL-6* in macrophage	Kurobe H [116]
Myriocin	Inhibitor of ceramide de novo synthesis pathway	BAPN	Reduce *MMPs* expression in macrophage	Yang H [86]
MCC950	Selective *NLRP3* inhibitor	High-fat/cholesterol diet + Ang II	Reduce *MMPs* expression in macrophage	Ren P [92]

TAAD, thoracic aortic aneurysm and dissection; BAPN, β-aminopropionitrile; Ang II, angiotensin II; *MR*, mineralocorticoid receptor; *MMP*, matrix metalloproteinase; *TNF*, tumor necrosis factor.

## 7. Conclusions

Activation of the inflammatory response and immune cell infiltration are essential hallmarks of TAAD. Macrophages play a pivotal role in the formation and progression of TAAD. Accumulation and infiltration of macrophages might drive inflammatory responses in the aortic media, produce *MMPs* to degrade ECM and further lead to thoracic aortic dilation, aneurysm, and dissection. Importantly, macrophages are regulated through accumulation, pro-inflammatory response, and ECM degradation. Targeting macrophages has promising translational value for preventing and treating TAAD in the future.

## Abbreviations

TAAD, thoracic aortic aneurysm and dissection; SMC, smooth muscle cell; ECM, extracellular matrix; EC, endothelial cell; BAPN, β-aminopropionitrile; NK Cell, natural killer cell; Ang II, angiotensin II; *MMP*, matrix metalloproteinase; *TIMP*, tissue inhibitors of metalloproteinases; *MHC*, major histocompatibility complex; *TNF*, tumor necrosis factor; *TGFBR*, transforming growth factor beta receptor; *TGFB*, transforming growth factor beta; *SMAD*, SMAD family member; *IL*, interleukin; *INF*, interferon; *CCL*, C-C motif chemokine ligand; *ADAMTS*, ADAM metallopeptidase with thrombospondin type 1 motif; *NLRP*, NLR family pyrin.

## References

[1] Isselbacher EM, Preventza O, Hamilton Black J 3rd, Augoustides JG, Beck AW, Bolen MA (2022). 2022 ACC/AHA Guideline for the Diagnosis and Management of Aortic Disease: A Report of the American Heart Association/American College of Cardiology Joint Committee on Clinical Practice Guidelines. *Circulation*.

[2] Isselbacher EM (2005). Thoracic and abdominal aortic aneurysms. *Circulation*.

[3] Pinard A, Jones GT, Milewicz DM (2019). Genetics of Thoracic and Abdominal Aortic Diseases. *Circulation Research*.

[4] Isselbacher EM, Lino Cardenas CL, Lindsay ME (2016). Hereditary Influence in Thoracic Aortic Aneurysm and Dissection. *Circulation*.

[5] Mahadevia R, Barker AJ, Schnell S, Entezari P, Kansal P, Fedak PWM (2014). Bicuspid aortic cusp fusion morphology alters aortic three-dimensional outflow patterns, wall shear stress, and expression of aortopathy. *Circulation*.

[6] Stock S, Mohamed SA, Sievers HH (2019). Bicuspid aortic valve related aortopathy. *General Thoracic and Cardiovascular Surgery*.

[7] del Porto F, Proietta M, Tritapepe L, Miraldi F, Koverech A, Cardelli P (2010). Inflammation and immune response in acute aortic dissection. *Annals of Medicine*.

[8] Elefteriades JA, Rizzo JA, Coady MA (1999). Thoracic aorta. *Radiology*.

[9] Tokgoz A, Wang S, Sastry P, Sun C, Figg NL, Huang Y (2022). Association of Collagen, Elastin, Glycosaminoglycans, and Macrophages with Tissue Ultimate Material Strength and Stretch in Human Thoracic Aortic Aneurysms: A Uniaxial Tension Study. *Journal of Biomechanical Engineering*.

[10] Postnov A, Suslov A, Sobenin I, Chairkin I, Sukhorukov V, Ekta MB (2021). Thoracic Aortic Aneurysm: Blood Pressure and Inflammation as Key Factors in the Development of Aneurysm Dissection. *Current Pharmaceutical Design*.

[11] Yang Y, Jiao X, Li L, Hu C, Zhang X, Pan L (2020). Increased Circulating Angiopoietin-Like Protein 8 Levels Are Associated with Thoracic Aortic Dissection and Higher Inflammatory Conditions. *Cardiovascular Drugs and Therapy*.

[12] Majesky MW, Dong XR, Hoglund VJ (2011). Parsing aortic aneurysms: more surprises. *Circulation Research*.

[13] Fan FD, Xu ZJ, Zhou Q, Wang DJ (2017). Expression profiles and clinical implication of plasma chemokines in patients with Stanford type A aortic dissection. *Zhonghua Xin Xue Guan Bing Za Zhi*.

[14] Luo F, Zhou XL, Li JJ, Hui RT (2009). Inflammatory response is associated with aortic dissection. *Ageing Research Reviews*.

[15] Shirasawa B, Hamano K, Kobayashi T, Kawamura T, Gohra H, Katoh T (2000). Could apoptosis be contributed to the occurrence of aortic dissection. *Kyobu Geka. The Japanese Journal of Thoracic Surgery*.

[16] Anidjar S, Dobrin PB, Eichorst M, Graham GP, Chejfec G (1992). Correlation of inflammatory infiltrate with the enlargement of experimental aortic aneurysms. *Journal of Vascular Surgery*.

[17] Dobrin PB, Baumgartner N, Anidjar S, Chejfec G, Mrkvicka R (1996). Inflammatory aspects of experimental aneurysms. Effect of methylprednisolone and cyclosporine. *Annals of the New York Academy of Sciences*.

[18] Gao H, Sun X, Liu Y, Liang S, Zhang B, Wang L (2021). Analysis of Hub Genes and the Mechanism of Immune Infiltration in Stanford Type a Aortic Dissection. *Frontiers in Cardiovascular Medicine*.

[19] Li Z, Wang J, Yu Q, Shen R, Qin K, Zhang Y (2022). Identification of Immune-Related Gene Signature in Stanford Type A Aortic Dissection. *Frontiers in Genetics*.

[20] Zhang L, Liao MF, Tian L, Zou SL, Lu QS, Bao JM (2011). Overexpression of interleukin-1β and interferon-γ in type I thoracic aortic dissections and ascending thoracic aortic aneurysms: possible correlation with matrix metalloproteinase-9 expression and apoptosis of aortic media cells. *European Journal of Cardio-thoracic Surgery: Official Journal of the European Association for Cardio-thoracic Surgery*.

[21] Xu Y, Ye J, Wang M, Wang Y, Ji Q, Huang Y (2018). Increased interleukin-11 levels in thoracic aorta and plasma from patients with acute thoracic aortic dissection. *Clinica Chimica Acta; International Journal of Clinical Chemistry*.

[22] Ye J, Wang M, Jiang H, Ji Q, Huang Y, Liu J (2018). Increased levels of interleukin-22 in thoracic aorta and plasma from patients with acute thoracic aortic dissection. *Clinica Chimica Acta; International Journal of Clinical Chemistry*.

[23] Kajander H, Paavonen T, Valo T, Tarkka M, Mennander AA (2013). Immunoglobulin G4-positive ascending thoracic aortitis may be prone to dissection. *The Journal of Thoracic and Cardiovascular Surgery*.

[24] Wu D, Choi JC, Sameri A, Minard CG, Coselli JS, Shen YH (2013). Inflammatory Cell Infiltrates in Acute and Chronic Thoracic Aortic Dissection. *Aorta (Stamford, Conn.)*.

[25] Lei C, Kan H, Chen W, Yang D, Ren J, Xu F (2022). Different gene co-expression patterns of aortic intima-media and adventitia in thoracic aortic aneurysm. *Gene*.

[26] He R, Guo DC, Estrera AL, Safi HJ, Huynh TT, Yin Z (2006). Characterization of the inflammatory and apoptotic cells in the aortas of patients with ascending thoracic aortic aneurysms and dissections. *The Journal of Thoracic and Cardiovascular Surgery*.

[27] Li Y, Ren P, Dawson A, Vasquez HG, Ageedi W, Zhang C (2020). Single-Cell Transcriptome Analysis Reveals Dynamic Cell Populations and Differential Gene Expression Patterns in Control and Aneurysmal Human Aortic Tissue. *Circulation*.

[28] Davis FM, Daugherty A, Lu HS (2019). Updates of Recent Aortic Aneurysm Research. *Arteriosclerosis, Thrombosis, and Vascular Biology*.

[29] Johnston WF, Salmon M, Pope NH, Meher A, Su G, Stone ML (2014). Inhibition of interleukin-1β decreases aneurysm formation and progression in a novel model of thoracic aortic aneurysms. *Circulation*.

[30] Ren W, Wang Z, Wang J, Wu Z, Ren Q, Yu A (2020). IL-5 overexpression attenuates aortic dissection by reducing inflammation and smooth muscle cell apoptosis. *Life Sciences*.

[31] Suehiro C, Suzuki J, Hamaguchi M, Takahashi K, Nagao T, Sakaue T (2019). Deletion of interleukin-18 attenuates abdominal aortic aneurysm formation. *Atherosclerosis*.

[32] Liu C, Zhang C, Jia L, Chen B, Liu L, Sun J (2018). Interleukin-3 stimulates matrix metalloproteinase 12 production from macrophages promoting thoracic aortic aneurysm/dissection. *Clinical Science (London, England: 1979)*.

[33] Ulndreaj A, Li A, Chen Y, Besla R, Pacheco S, Althagafi MG (2021). Adventitial recruitment of Lyve-1- macrophages drives aortic aneurysm in an angiotensin-2-based murine model. *Clinical Science (London, England: 1979)*.

[34] Liu X, Chen W, Zhu G, Yang H, Li W, Luo M (2022). Single-cell RNA sequencing identifies an Il1rn^+^/Trem1^+^ macrophage subpopulation as a cellular target for mitigating the progression of thoracic aortic aneurysm and dissection. *Cell Discovery*.

[35] Ju X, Ijaz T, Sun H, Ray S, Lejeune W, Lee C (2013). Interleukin-6-signal transducer and activator of transcription-3 signaling mediates aortic dissections induced by angiotensin II via the T-helper lymphocyte 17-interleukin 17 axis in C57BL/6 mice. *Arteriosclerosis, Thrombosis, and Vascular Biology*.

[36] Suen RS, Rampersad SN, Stewart DJ, Courtman DW (2011). Differential roles of endothelin-1 in angiotensin II-induced atherosclerosis and aortic aneurysms in apolipoprotein E-null mice. *The American Journal of Pathology*.

[37] Harris D, Liang Y, Chen C, Li S, Patel O, Qin Z (2015). Bone marrow from blotchy mice is dispensable to regulate blood copper and aortic pathologies but required for inflammatory mediator production in LDLR-deficient mice during chronic angiotensin II infusion. *Annals of Vascular Surgery*.

[38] Li X, Liu D, Zhao L, Wang L, Li Y, Cho K (2020). Targeted depletion of monocyte/macrophage suppresses aortic dissection with the spatial regulation of MMP-9 in the aorta. *Life Sciences*.

[39] van der Vorst EPC, Weber C (2019). Novel Features of Monocytes and Macrophages in Cardiovascular Biology and Disease. *Arteriosclerosis, Thrombosis, and Vascular Biology*.

[40] Xu L, Burke A (2013). Acute medial dissection of the ascending aorta: evolution of reactive histologic changes. *The American Journal of Surgical Pathology*.

[41] Perdiguero EG, Geissmann F (2016). The development and maintenance of resident macrophages. *Nature Immunology*.

[42] Ginhoux F, Guilliams M (2016). Tissue-Resident Macrophage Ontogeny and Homeostasis. *Immunity*.

[43] Michineau S, Franck G, Wagner-Ballon O, Dai J, Allaire E, Gervais M (2014). Chemokine (C-X-C motif) receptor 4 blockade by AMD3100 inhibits experimental abdominal aortic aneurysm expansion through anti-inflammatory effects. *Arteriosclerosis, Thrombosis, and Vascular Biology*.

[44] Shen YH, Hu X, Zou S, Wu D, Coselli JS, LeMaire SA (2012). Stem cells in thoracic aortic aneurysms and dissections: potential contributors to aortic repair. *The Annals of Thoracic Surgery*.

[45] Yamawaki-Ogata A, Hashizume R, Fu XM, Usui A, Narita Y (2014). Mesenchymal stem cells for treatment of aortic aneurysms. *World Journal of Stem Cells*.

[46] Murray PJ, Allen JE, Biswas SK, Fisher EA, Gilroy DW, Goerdt S (2014). Macrophage activation and polarization: nomenclature and experimental guidelines. *Immunity*.

[47] Liu Y, Zou L, Tang H, Li J, Liu H, Jiang X (2022). Single-Cell Sequencing of Immune Cells in Human Aortic Dissection Tissue Provides Insights Into Immune Cell Heterogeneity. *Frontiers in Cardiovascular Medicine*.

[48] Hänzelmann S, Castelo R, Guinney J (2013). GSVA: gene set variation analysis for microarray and RNA-seq data. *BMC Bioinformatics*.

[49] Cochain C, Vafadarnejad E, Arampatzi P, Pelisek J, Winkels H, Ley K (2018). Single-Cell RNA-Seq Reveals the Transcriptional Landscape and Heterogeneity of Aortic Macrophages in Murine Atherosclerosis. *Circulation Research*.

[50] Hashimoto D, Chow A, Noizat C, Teo P, Beasley MB, Leboeuf M (2013). Tissue-resident macrophages self-maintain locally throughout adult life with minimal contribution from circulating monocytes. *Immunity*.

[51] Maiellaro K, Taylor WR (2007). The role of the adventitia in vascular inflammation. *Cardiovascular Research*.

[52] Mellak S, Ait-Oufella H, Esposito B, Loyer X, Poirier M, Tedder TF (2015). Angiotensin II mobilizes spleen monocytes to promote the development of abdominal aortic aneurysm in Apoe-/- mice. *Arteriosclerosis, Thrombosis, and Vascular Biology*.

[53] Nahrendorf M, Keliher E, Marinelli B, Leuschner F, Robbins CS, Gerszten RE (2011). Detection of macrophages in aortic aneurysms by nanoparticle positron emission tomography-computed tomography. *Arteriosclerosis, Thrombosis, and Vascular Biology*.

[54] Tieu BC, Ju X, Lee C, Sun H, Lejeune W, Recinos A 3rd (2011). Aortic adventitial fibroblasts participate in angiotensin-induced vascular wall inflammation and remodeling. *Journal of Vascular Research*.

[55] Le S, Zhang H, Huang X, Chen S, Wu J, Chen S (2020). PKM2 Activator TEPP-46 Attenuates Thoracic Aortic Aneurysm and Dissection by Inhibiting NLRP3 Inflammasome-Mediated IL-1β Secretion. *Journal of Cardiovascular Pharmacology and Therapeutics*.

[56] Pisano C, Balistreri CR, Ricasoli A, Ruvolo G (2017). Cardiovascular Disease in Ageing: An Overview on Thoracic Aortic Aneurysm as an Emerging Inflammatory Disease. *Mediators of Inflammation*.

[57] Wang Q, Guo X, Huo B, Feng X, Fang ZM, Jiang DS (2022). Integrating Bulk Transcriptome and Single-Cell RNA Sequencing Data Reveals the Landscape of the Immune Microenvironment in Thoracic Aortic Aneurysms. *Frontiers in Cardiovascular Medicine*.

[58] Song W, Qin L, Chen Y, Chen J, Wei L (2023). Single-cell transcriptome analysis identifies Versican(+) myofibroblast as a hallmark for thoracic aortic aneurysm marked by activation of PI3K-AKT signaling pathway. *Biochemical and Biophysical Research Communications*.

[59] Arango Duque G, Descoteaux A (2014). Macrophage cytokines: involvement in immunity and infectious diseases. *Frontiers in Immunology*.

[60] Mosser DM, Edwards JP (2008). Exploring the full spectrum of macrophage activation. *Nature Reviews Immunology*.

[61] Khoury MK, Yang H, Liu B (2021). Macrophage Biology in Cardiovascular Diseases. *Arteriosclerosis, Thrombosis, and Vascular Biology*.

[62] Anzai A, Shimoda M, Endo J, Kohno T, Katsumata Y, Matsuhashi T (2015). Adventitial CXCL1/G-CSF expression in response to acute aortic dissection triggers local neutrophil recruitment and activation leading to aortic rupture. *Circulation Research*.

[63] Tieu BC, Lee C, Sun H, Lejeune W, Recinos A 3rd, Ju X (2009). An adventitial IL-6/MCP1 amplification loop accelerates macrophage-mediated vascular inflammation leading to aortic dissection in mice. *The Journal of Clinical Investigation*.

[64] Bendeck MP (2002). Matrix metalloproteinases: are they antiatherogenic but proaneurysmal. *Circulation Research*.

[65] Luo W, Wang Y, Zhang L, Ren P, Zhang C, Li Y (2020). Critical Role of Cytosolic DNA and Its Sensing Adaptor STING in Aortic Degeneration, Dissection, and Rupture. *Circulation*.

[66] Lepidi S, Kenagy RD, Raines EW, Chiu ES, Chait A, Ross R (2001). MMP9 production by human monocyte-derived macrophages is decreased on polymerized type I collagen. *Journal of Vascular Surgery*.

[67] Faisal Khan KM, Laurie GW, McCaffrey TA, Falcone DJ (2002). Exposure of cryptic domains in the alpha 1-chain of laminin-1 by elastase stimulates macrophages urokinase and matrix metalloproteinase-9 expression. *The Journal of Biological Chemistry*.

[68] Xu JM, Shi GP (2012). Emerging role of mast cells and macrophages in cardiovascular and metabolic diseases. *Endocrine Reviews*.

[69] Moehle CW, Bhamidipati CM, Alexander MR, Mehta GS, Irvine JN, Salmon M (2011). Bone marrow-derived MCP1 required for experimental aortic aneurysm formation and smooth muscle phenotypic modulation. *The Journal of Thoracic and Cardiovascular Surgery*.

[70] Raffort J, Lareyre F, Clément M, Hassen-Khodja R, Chinetti G, Mallat Z (2017). Monocytes and macrophages in abdominal aortic aneurysm. *Nature Reviews. Cardiology*.

[71] Ye P, Chen W, Wu J, Huang X, Li J, Wang S (2013). GM-CSF contributes to aortic aneurysms resulting from SMAD3 deficiency. *The Journal of Clinical Investigation*.

[72] Ren P, Hughes M, Krishnamoorthy S, Zou S, Zhang L, Wu D (2017). Critical Role of ADAMTS-4 in the Development of Sporadic Aortic Aneurysm and Dissection in Mice. *Scientific Reports*.

[73] Williams H, Wadey KS, Frankow A, Blythe HC, Forbes T, Johnson JL (2021). Aneurysm severity is suppressed by deletion of CCN4. *Journal of Cell Communication and Signaling*.

[74] Hawkins RB, Salmon M, Su G, Lu G, Leroy V, Bontha SV (2021). Mesenchymal Stem Cells Alter MicroRNA Expression and Attenuate Thoracic Aortic Aneurysm Formation. *The Journal of Surgical Research*.

[75] Chou EL, Chaffin M, Simonson B, Pirruccello JP, Akkad AD, Nekoui M (2022). Aortic Cellular Diversity and Quantitative Genome-Wide Association Study Trait Prioritization Through Single-Nuclear RNA Sequencing of the Aneurysmal Human Aorta. *Arteriosclerosis, Thrombosis, and Vascular Biology*.

[76] Yang G, Zhang J, Jiang T, Monslow J, Tang SY, Todd L (2020). Bmal1 Deletion in Myeloid Cells Attenuates Atherosclerotic Lesion Development and Restrains Abdominal Aortic Aneurysm Formation in Hyperlipidemic Mice. *Arteriosclerosis, Thrombosis, and Vascular Biology*.

[77] Ren P, Zhang L, Xu G, Palmero LC, Albini PT, Coselli JS (2013). ADAMTS-1 and ADAMTS-4 levels are elevated in thoracic aortic aneurysms and dissections. *The Annals of Thoracic Surgery*.

[78] Wang X, Zhang H, Ge Y, Cao L, He Y, Sun G (2021). AT1R Regulates Macrophage Polarization Through YAP and Regulates Aortic Dissection Incidence. *Frontiers in Physiology*.

[79] Hara H, Maemura S, Fujiwara T, Takeda N, Ishii S, Yagi H (2020). Inhibition of transforming growth factor-β signaling in myeloid cells ameliorates aortic aneurysmal formation in Marfan syndrome. *PloS One*.

[80] Wang Q, Chen Z, Peng X, Zheng Z, Le A, Guo J (2021). Neuraminidase 1 Exacerbating Aortic Dissection by Governing a Pro-Inflammatory Program in Macrophages. *Frontiers in Cardiovascular Medicine*.

[81] Wang S, Liu Y, Zhao G, He L, Fu Y, Yu C (2018). Postnatal deficiency of ADAMTS1 ameliorates thoracic aortic aneurysm and dissection in mice. *Experimental Physiology*.

[82] Zhang Z, Jiang Y, Zhou Z, Huang J, Chen S, Zhou W (2019). Scavenger receptor A1 attenuates aortic dissection via promoting efferocytosis in macrophages. *Biochemical Pharmacology*.

[83] Aoki H, Majima R, Hashimoto Y, Hirakata S, Ohno-Urabe S (2021). Ying and Yang of Stat3 in pathogenesis of aortic dissection. *Journal of Cardiology*.

[84] Ohno-Urabe S, Aoki H, Nishihara M, Furusho A, Hirakata S, Nishida N (2018). Role of Macrophage Socs3 in the Pathogenesis of Aortic Dissection. *Journal of the American Heart Association*.

[85] Patel J, McNeill E, Douglas G, Hale AB, de Bono J, Lee R (2015). RGS1 regulates myeloid cell accumulation in atherosclerosis and aortic aneurysm rupture through altered chemokine signalling. *Nature Communications*.

[86] Yang H, Yang F, Luo M, Chen Q, Liu X, Zhang Y (2022). Metabolomic Profile Reveals That Ceramide Metabolic Disturbance Plays an Important Role in Thoracic Aortic Dissection. *Frontiers in Cardiovascular Medicine*.

[87] Cui H, Chen Y, Li K, Zhan R, Zhao M, Xu Y (2021). Untargeted metabolomics identifies succinate as a biomarker and therapeutic target in aortic aneurysm and dissection. *European Heart Journal*.

[88] Nishimura M, Yamashita A, Matsuura Y, Okutsu J, Fukahori A, Hirata T (2021). Upregulated Kynurenine Pathway Enzymes in Aortic Atherosclerotic Aneurysm: Macrophage Kynureninase Downregulates Inflammation. *Journal of Atherosclerosis and Thrombosis*.

[89] Pan L, Bai P, Weng X, Liu J, Chen Y, Chen S (2022). Legumain Is an Endogenous Modulator of Integrin αvβ3 Triggering Vascular Degeneration, Dissection, and Rupture. *Circulation*.

[90] Carmeliet P, Moons L, Lijnen R, Baes M, Lemaître V, Tipping P (1997). Urokinase-generated plasmin activates matrix metalloproteinases during aneurysm formation. *Nature Genetics*.

[91] Lian G, Li X, Zhang L, Zhang Y, Sun L, Zhang X (2019). Macrophage metabolic reprogramming aggravates aortic dissection through the HIF1α-ADAM17 pathway. *EBioMedicine*.

[92] Ren P, Wu D, Appel R, Zhang L, Zhang C, Luo W (2020). Targeting the NLRP3 Inflammasome With Inhibitor MCC950 Prevents Aortic Aneurysms and Dissections in Mice. *Journal of the American Heart Association*.

[93] Liao M, Zou S, Bao Y, Jin J, Yang J, Liu Y (2018). Matrix metalloproteinases are regulated by MicroRNA 320 in macrophages and are associated with aortic dissection. *Experimental Cell Research*.

[94] Albini PT, Segura AM, Liu G, Minard CG, Coselli JS, Milewicz DM (2014). Advanced atherosclerosis is associated with increased medial degeneration in sporadic ascending aortic aneurysms. *Atherosclerosis*.

[95] Tanaka H, Iida Y, Iwaki T, Suzuki Y, Sano H, Miyajima C (2018). Elevated Plasma Levels of LDL Cholesterol Promote Dissecting Thoracic Aortic Aneurysms in Angiotensin II-Induced Mice. *Annals of Vascular Surgery*.

[96] Chun C, Qi X, Wang F, Madrid KB, Saldarriaga LA, Fisch MR (2021). Nicotine Exacerbates TAAD Formation Induced by Smooth Muscle-Specific Deletion of the TGF-β Receptor 2. *Journal of Immunology Research*.

[97] Landenhed M, Engström G, Gottsäter A, Caulfield MP, Hedblad B, Newton-Cheh C (2015). Risk profiles for aortic dissection and ruptured or surgically treated aneurysms: a prospective cohort study. *Journal of the American Heart Association*.

[98] Ishibashi M, Egashira K, Zhao Q, Hiasa KI, Ohtani K, Ihara Y (2004). Bone marrow-derived monocyte chemoattractant protein-1 receptor CCR2 is critical in angiotensin II-induced acceleration of atherosclerosis and aneurysm formation in hypercholesterolemic mice. *Arteriosclerosis, Thrombosis, and Vascular Biology*.

[99] Combadière C, Potteaux S, Rodero M, Simon T, Pezard A, Esposito B (2008). Combined inhibition of CCL2, CX3CR1, and CCR5 abrogates Ly6C(hi) and Ly6C(lo) monocytosis and almost abolishes atherosclerosis in hypercholesterolemic mice. *Circulation*.

[100] Ley K (2003). The role of selectins in inflammation and disease. *Trends in Molecular Medicine*.

[101] Hannawa KK, Eliason JL, Woodrum DT, Pearce CG, Roelofs KJ, Grigoryants V (2005). L-selectin-mediated neutrophil recruitment in experimental rodent aneurysm formation. *Circulation*.

[102] Soehnlein O, Lindbom L, Weber C (2009). Mechanisms underlying neutrophil-mediated monocyte recruitment. *Blood*.

[103] Davis FM, Gallagher KA (2019). Epigenetic Mechanisms in Monocytes/Macrophages Regulate Inflammation in Cardiometabolic and Vascular Disease. *Arteriosclerosis, Thrombosis, and Vascular Biology*.

[104] Akita N, Narita Y, Yamawaki-Ogata A, Usui A, Komori K (2021). Therapeutic effect of allogeneic bone marrow-derived mesenchymal stromal cells on aortic aneurysms. *Cell and Tissue Research*.

[105] Yamawaki-Ogata A, Fu X, Hashizume R, Fujimoto KL, Araki Y, Oshima H (2014). Therapeutic potential of bone marrow-derived mesenchymal stem cells in formed aortic aneurysms of a mouse model. *European Journal of Cardio-thoracic Surgery: Official Journal of the European Association for Cardio-thoracic Surgery*.

[106] Kozakai M, Narita Y, Yamawaki-Ogata A, Fujimoto KL, Mutsuga M, Tokuda Y (2022). Alternative therapeutic strategy for existing aortic aneurysms using mesenchymal stem cell-derived exosomes. *Expert Opinion on Biological Therapy*.

[107] Kurobe H, Matsuoka Y, Hirata Y, Sugasawa N, Maxfield MW, Sata M (2013). Azelnidipine suppresses the progression of aortic aneurysm in wild mice model through anti-inflammatory effects. *The Journal of Thoracic and Cardiovascular Surgery*.

[108] Hibino M, Otaki Y, Kobeissi E, Pan H, Hibino H, Taddese H (2022). Blood Pressure, Hypertension, and the Risk of Aortic Dissection Incidence and Mortality: Results from the J-SCH Study, the UK Biobank Study, and a Meta-Analysis of Cohort Studies. *Circulation*.

[109] Mulè G, Nardi E, Morreale M, Castiglia A, Geraci G, Altieri D (2017). The Relationship Between Aortic Root Size and Hypertension: An Unsolved Conundrum. *Advances in Experimental Medicine and Biology*.

[110] Milutinović A, Zorc-Pleskovič R (2022). Inflammatory cells in the ascending aortic aneurysm in patients with type 2 diabetes versus patients with hypertension. *Bosnian Journal of Basic Medical Sciences*.

[111] Cheng Z, Zhou YZ, Wu Y, Wu QY, Liao XB, Fu XM (2018). Diverse roles of macrophage polarization in aortic aneurysm: destruction and repair. *Journal of Translational Medicine*.

[112] Zhu L, Zhao Q, Yang T, Ding W, Zhao Y (2015). Cellular metabolism and macrophage functional polarization. *International Reviews of Immunology*.

[113] Yan J, Horng T (2020). Lipid Metabolism in Regulation of Macrophage Functions. *Trends in Cell Biology*.

[114] Saha S, Shalova IN, Biswas SK (2017). Metabolic regulation of macrophage phenotype and function. *Immunological Reviews*.

[115] Wang X, Zhang X, Qiu T, Yang Y, Li Q, Zhang X (2021). Dexamethasone reduces the formation of thoracic aortic aneurysm and dissection in a murine model. *Experimental Cell Research*.

[116] Kurobe H, Hirata Y, Matsuoka Y, Sugasawa N, Higashida M, Nakayama T (2013). Protective effects of selective mineralocorticoid receptor antagonist against aortic aneurysm progression in a novel murine model. *The Journal of Surgical Research*.

[117] Ye D, Wu C, Chen H, Liang CL, Howatt DA, Franklin MK (2022). Fludrocortisone Induces Aortic Pathologies in Mice. *Biomolecules*.

[118] Reilly JM, Savage EB, Brophy CM, Tilson MD (1990). Hydrocortisone rapidly induces aortic rupture in a genetically susceptible mouse. *Archives of Surgery (Chicago, Ill.: 1960)*.

[119] Libby P (2018). Biologically-Based Therapies for Aortic Diseases: Why the Long Lag in Translation. *Journal of the American College of Cardiology*.

